# Inflammation mediates the association between muscle mass and accelerated phenotypic aging: results from the NHANES 2011–2018

**DOI:** 10.3389/fnut.2024.1503702

**Published:** 2025-01-06

**Authors:** Shifu Bao, Weibu Jimu, Nai Mu, Fang Yan, Shuxing Xing, Tao Li, Zheng Zhou

**Affiliations:** ^1^Department of Medicine and Life Sciences, Chengdu University of Traditional Chinese Medicine, Chengdu, China; ^2^Department of Orthopedics, Chengdu Fifth People's Hospital, Chengdu, China; ^3^Department of Geriatrics, Chengdu Fifth People's Hospital, Chengdu, China; ^4^Department of Orthopedic Surgery and Orthopedic Research Institute, West China Hospital, Sichuan University, Chengdu, China

**Keywords:** PhenoAge, skeletal muscle, aging, SII, NHANES, cross-sectional study

## Abstract

**Background:**

Muscle mass plays a pivotal role in health maintenance, yet its connection to biological aging remains underexplored. This study investigates the association between appendicular skeletal muscle mass index (ASMI) and phenotypic age(PhenoAge), while examining the mediating role of systemic inflammation.

**Methods:**

The analysis included 7,440 participants from the NHANES 2011–2018. Phenotypic Age Acceleration (PhenoAgeAccel) was calculated as the residuals from regressing PhenoAge on chronological age. Multivariable linear regression analyses were performed to assess the association between ASMI and PhenoAgeAccel. Mediation analysis was conducted to quantify the extent to which systemic inflammation contributes to this association.

**Results:**

Our analysis revealed that higher ASMI is linked to slower biological aging, as evidenced by lower PhenoAgeAccel (*β* = −0.48, 95% CI: −0.66 to −0.29, *p* = 0.0001). Systemic inflammation partially mediated this effect, with a mediation proportion of 35.1%. The association varied notably across demographic and health-related subgroups, being particularly significant in females, individuals with obesity, and those with lower physical activity.

**Conclusion:**

These findings highlight the critical importance of muscle mass in slowing biological aging, with systemic inflammation emerging as a key biological mediator. The public health implications are substantial, suggesting that targeted interventions—such as resistance training, anti-inflammatory diets, and personalized medical approaches—could play a pivotal role in decelerating biological aging and improving long-term health outcomes.

## 1 Introduction

Aging is a universal biological process that everyone undergoes. With age, bodily functions gradually decline, leading to increased susceptibility to disease. By 2030, nearly one-sixth of the global population will be 60 years or older. While chronological age is a major indicator of mortality risk, it mainly reflects the passage of time, providing limited insight into an individual’s health or the rate of aging (1). In contrast, phenotypic age (PhenoAge), derived from clinical biomarkers, more accurately assesses biological age and better predicts risks related to age-associated diseases (2, 3). Phenotypic Age Acceleration (PhenoAgeAccel), measuring the gap between biological and chronological age, is widely used in health and longevity studies (4, 5). Positive PhenoAgeAccel values signal accelerated aging and heightened health risks (6). This metric is valuable in research on environmentally induced aging, as it helps identify high-risk groups and inform targeted interventions.

Muscle mass is essential for health and physical function, yet it significantly declines with age. In young adults, lean muscle constitutes about 50% of body weight. By ages 70 to 80, it accounts for only 25%. This loss reduces muscle strength and endurance, hindering daily activities and quality of life (7–10). Studies have shown that muscle strength and mass begin to decline as early as the third or fourth decade (11–14). Greater muscle mass has been associated with delayed onset of diseases like obesity, type 2 diabetes, cardiovascular disease, stroke, and some cancers (15). Although muscle mass reduction has been linked to the telomere shortening (16), the link between muscle mass and biological aging remains underexplored. Further research is needed to understand the relationship between appendicular skeletal muscle mass index (ASMI) and phenotypic age.

Chronic inflammation is a hallmark of aging and a recognized indicator of the aging process. Evidence shows that aging involves elevated levels of pro-inflammatory cytokines and increased production of these cytokines by cells. Higher levels of cytokines such as IL-6, TNF-*α*, and CRP, along with increased leukocyte counts, have been associated with aging (17, 18). Prolonged exposure to inflammatory markers like CRP, IL-6, and TNF-α can promote muscle atrophy by disrupting anabolic processes and energy homeostasis (19). Lifestyle interventions, such as physical activity and dietary changes, may reduce inflammation and improve muscle mass (20). We hypothesize that inflammatory responses mediate the relationship between muscle mass and PhenoAgeAccel, offering key insights into the mechanisms at play.

This study uses data from the National Health and Nutrition Examination Survey (NHANES) to explore the relationship between ASMI and PhenoAgeAccel, and to investigate whether this relationship is mediated by the Systemic Inflammation Index (SII).

## 2 Methods

**Figure 1:**
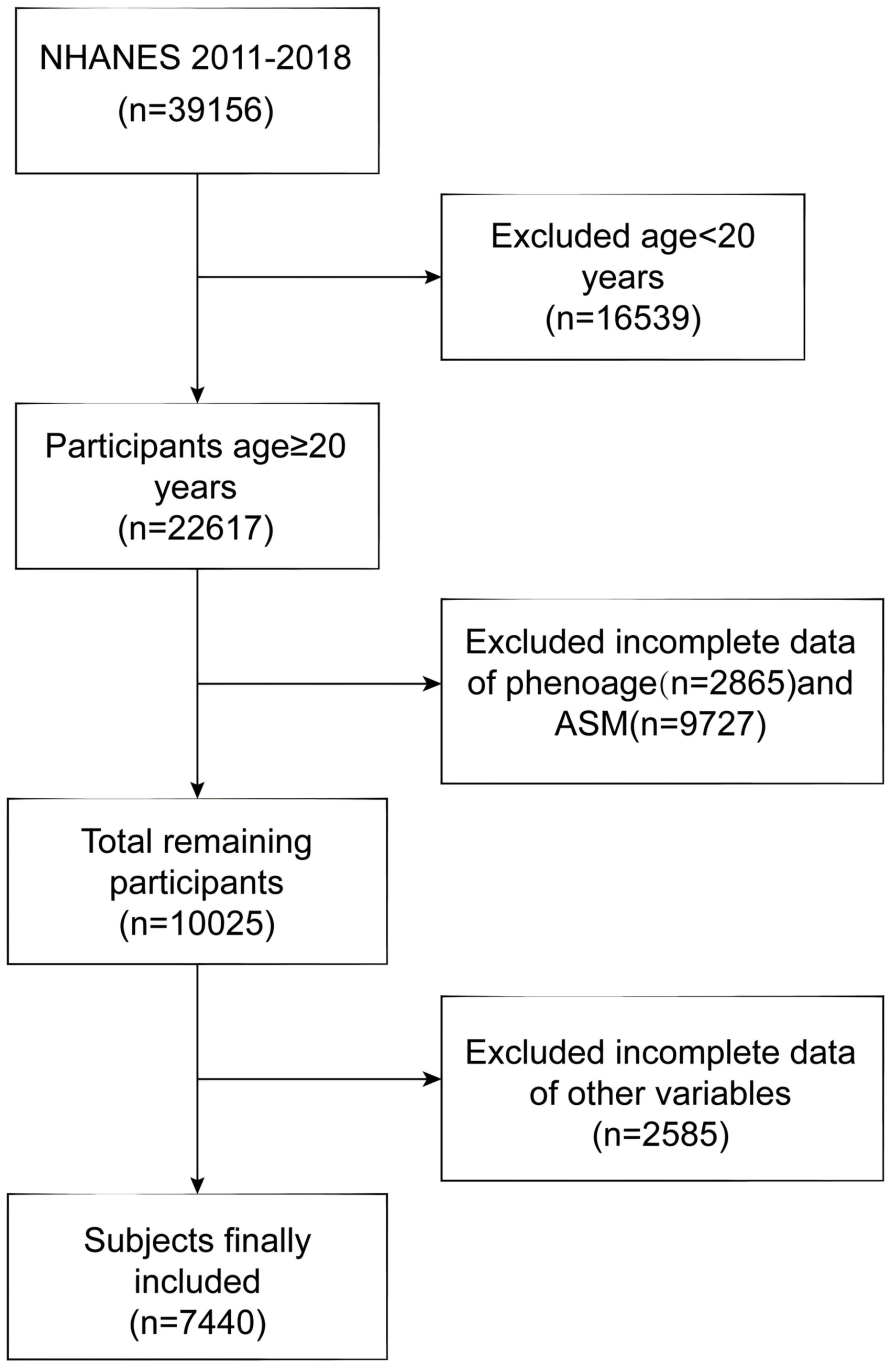
Flowchart of the sample selection from NHANES 2011–2018.

This cross-sectional study utilized data from the U.S. National Health and Nutrition Examination Survey,^1^ which offers a nationally representative assessment of the U.S. population’s health and nutritional status. The study protocol was approved by the Ethics Review Board of the National Center for Health Statistics, with the latest approval in August 2022. All participants provided written informed consent. Data from the 2011–2018 NHANES cycles were analyzed, spanning four survey periods. Of the initial 39,156 participants, exclusions were made for those under 20 years old (*n* = 16,539), missing PhenoAge data (*n* = 2,865), missing ASM data (*n* = 9,727), and those with incomplete data for other variables (*n* = 2,585). The final cohort consisted of 7,440 individuals aged 20 years and older. A detailed flow of participant selection is illustrated in Figure 1.

### 2.1 Muscle mass

Appendicular skeletal muscle mass (ASM) was assessed using dual-energy X-ray absorptiometry (DXA), a widely validated method for measuring lean soft tissue mass in both arms and legs. ASM was calculated by summing the lean tissue mass of the limbs. To further assess muscle mass, the ASMI was derived by dividing ASM by height squared (ASM/height^2^) (21, 22). ASMI values were categorized into quartiles based on distribution: ≤6.82 for quartile 1, 6.83–8.01 for quartile 2, 8.02–9.21 for quartile 3, and ≥ 9.22 for quartile 4.

### 2.2 PhenoAge

PhenoAge is calculated using nine clinical biomarkers that represent key physiological systems associated with aging and inflammation: albumin (indicator of liver function), alkaline phosphatase (marker of liver and bone metabolism), creatinine (reflecting kidney function), HbA1c (representing metabolic health), C-reactive protein (CRP; a marker of inflammation), lymphocyte percentage (indicator of immune function), mean cell volume (MCV; related to immune function), red cell distribution width (RDW; associated with immune function), and white blood cell count (WBC; indicative of inflammation and immune function). Together, these biomarkers provide a comprehensive reflection of systemic health and are intricately linked to the biological aging process (23). In this study, we utilized the BioAge R package to train the PhenoAge model on NHANES III data, applying it to NHANES IV data (1999–2018). We excluded CRP due to its unavailability in the NHANES 2011–2018 cycles. However, a comparison of PhenoAge values with and without CRP in the 1999–2010 dataset demonstrated a strong correlation (0.959–0.996) (24), this finding underscores the robustness of the PhenoAge model, suggesting that CRP, while a widely recognized marker of systemic inflammation, may not be essential for capturing the overall effects of chronic inflammation on biological aging. Importantly, the exclusion of CRP also minimizes the potential confounding influence of acute inflammatory events, ensuring that the model remains focused on chronic inflammation, which is more relevant to the context of aging. PhenoAgeAccel was quantified as the residual from a linear regression of PhenoAge on chronological age, with values >0 indicating accelerated aging and values <0 signifying younger phenotypic age (25).

### 2.3 SII

The Systemic Immune-Inflammation Index is a marker used to assess inflammatory status. It is calculated using blood counts of neutrophils, lymphocytes, and platelets, following the formula: SII = platelet count × neutrophil count / lymphocyte count (26, 27).

### 2.4 Covariates

Referring to the prior research and clinical insights, we accounted for covariates that could potentially impact the link between muscle mass and PhenoAge. The covariates in this study included age (year), gender (male/female), ethnicity (American/White/Black/other), marital status (married/living with a partner and widowed/divorced/separated/never married), education attainment (Less than 12th grade, high school graduate or equivalent, some college or AA degree, and college graduate or above), poverty income ratio (PIR), smoking status (never, former, and now), drinking status (never, former, mild, moderate, heavy), physical activity (PA, yes/no), hypertension (yes/no), diabetes (yes/ no), Cardiovascular diseases (CVD, yes/ no), body mass index (BMI, kg/m2), total protein (g/dL), blood urea nitrogen (mg/dL), serum creatinine (mg/dL), serum uric acid (mg/dL), serum calcium (mg/dL), alkaline phosphatase (ALP, u/L) and serum phosphorus (mg/dL). Details of each variable were publicly available at www.cdc.gov/nchs/nhanes/.

### 2.5 Statistical analysis

Following Centers for Disease Control and Prevention (CDC) guidelines, statistical analyses utilized NHANES sampling weights, recalculated per National Center for Health Statistics (NCHS) recommendations to account for the survey’s complex design. Categorical variables were analyzed using weighted chi-square tests, while continuous variables were assessed via one-way analysis of variance (ANOVA). The relationship between ASMI and PhenoAgeAccel was explored through multivariable linear regression across three models. Model 1 was unadjusted; Model 2 adjusted for gender, age, and race; Model 3 included additional adjustments for marital status, education, PIR, smoking status, drinking status, PA, hypertension, diabetes, CVD, BMI, and biochemical markers (total protein, blood urea nitrogen, serum creatinine, serum uric acid, serum calcium, alkaline phosphatase, and serum phosphorus). Due to its non-normal distribution, SII was log2-transformed for regression analyses. Subgroup analyses stratified participants by age (<45, ≥45 years), gender(male, female), race (White, Black, Mexican American, others), PIR (<1, 1–3, >3), PA (no, yes), smoking status (never, former, current), BMI (<25, 25–29.9, >29.9 kg/m2), hypertension, and diabetes. These stratification factors were also examined as potential effect modifiers. To comprehensively explore causal pathways and provide actionable insights for clinical practice, we employed mediation analysis to investigate how inflammation potentially mediates the relationship between ASMI and PhenoAgeAccel. Mediation analyses, conducted using the ‘mediation’ package in R (version 4.1.3) with 5,000 bootstraps, assessed SII’s role as a mediator in the ASMI-PhenoAgeAccel relationship. Direct effects reflected ASMI’s influence on PhenoAgeAccel, while indirect effects quantified SII’s mediating role. The proportion mediated was determined by dividing the indirect effect by the total effect. Restricted cubic spline (RCS) models were used to investigate the potential dose–response relationships between ASMI and age, PhenoAgeAccel, and SII, respectively.

Data processing and statistical analyses were performed in R version 4.1.3, with significance defined at *p* < 0.05.

## 3 Results

### 3.1 Baseline characteristics of participants

Table 1 summarizes the baseline characteristics of the 7,440 participants, stratified by ASMI quartiles. The sample comprised 53.06% males and 46.94% females, with an average age of 38.70 ± 0.30 years. Significant differences were observed across ASMI quartiles in several demographic and health-related variables, including age, gender, race, PIR, BMI, education, marital status, smoking status, drinking status, physical activity, PhenoAge, serum uric acid, blood urea nitrogen, alkaline phosphatase, and serum phosphorus (all *p*-values <0.05). Participants in the higher ASMI quartiles were more likely to be male, non-smokers, and had higher BMI, ALP, serum creatinine, serum uric acid, and blood urea nitrogen levels. They also tended to have higher educational attainment and engage in more physical activity, suggesting a correlation between higher ASMI and healthier lifestyle behaviors and physiological markers.

**Table 1:** Characteristics of the study participants according to ASMI levels.

Characteristic	Total (*n* = 7,440)	Q1 (*n* = 1859)	Q2 (*n* = 1861)	Q3 (*n* = 1858)	Q4 (*n* = 1862)	*P* value
Age(years)	38.70 ± 0.30	39.33 ± 0.49	38.08 ± 0.47	39.19 ± 0.35	38.13 ± 0.44	0.01
Gender %						< 0.0001
Female	3,515 (46.94)	1,698 (92.23)	976 (51.21)	505 (23.58)	336 (14.94)	
Male	3,925 (53.06)	161(7.77)	885 (48.79)	1,353 (76.42)	1,526 (85.06)	
Ethnicity %						< 0.0001
Black	1,512 (10.33)	169(4.13)	297(8.25)	397 (11.01)	649 (18.90)	
Mexican American	1,063(9.85)	225(7.15)	263(9.32)	288 (11.05)	287 (12.24)	
Other	2079 (15.81)	653 (17.22)	559 (16.48)	487 (15.14)	380 (14.19)	
White	2,786 (64.01)	812 (71.50)	742 (65.95)	686 (62.81)	546 (54.66)	
Education %						< 0.0001
Less than 12th grade	1,132(10.65)	226 (8.43)	310 (11.45)	323 (12.63)	273 (10.25)	
High school grade or equivalent	1,599 (21.07)	325 (16.65)	388 (21.05)	426 (22.81)	460 (24.35)	
Some college or AA degree	2,516 (33.61)	613 (32.92)	602 (33.59)	594 (31.42)	707 (36.71)	
College graduate or above	2,193 (34.67)	695 (42.00)	561 (33.91)	515 (33.14)	422 (28.69)	
Marital status %						0.02
Divorced	666(9.01)	181 (10.06)	178(9.74)	174(9.16)	133(6.89)	
Living with partner	836 (10.65)	180(9.34)	210 (11.64)	222 (11.29)	224 (10.44)	
Married	3,548 (51.08)	918 (52.82)	845 (46.55)	889 (51.35)	896 (53.53)	
Never married	2063 (25.77)	492 (24.11)	532 (28.26)	490 (24.62)	549 (26.27)	
Separated	242(2.57)	59 (2.44)	61 (2.49)	69 (2.90)	53 (2.47)	
Widowed	85(0.92)	29 (1.23)	35 (1.33)	14 (0.69)	7 (0.39)	
Smoking status %						< 0.001
Never	4,516 (59.21)	1,266 (63.57)	1,101 (57.17)	1,031 (55.35)	1,118 (60.37)	
Former	1,277 (19.94)	240 (16.23)	299 (18.88)	379 (23.08)	359 (22.03)	
Now	1,647 (20.85)	353 (20.21)	461 (23.95)	448 (21.57)	385 (17.60)	
Drinking status %						< 0.0001
Never	885(9.06)	298 (10.78)	236(9.81)	177(7.40)	174(8.03)	
Former	637(7.72)	135 (6.23)	157 (7.28)	184 (9.47)	161 (8.04)	
Mild	2,504 (34.41)	556 (30.88)	616 (33.60)	667 (37.02)	665 (36.59)	
Moderate	1,427 (20.72)	469 (29.46)	337 (19.50)	329 (17.76)	292 (15.11)	
Heavy	1987 (28.09)	401 (22.66)	515 (29.81)	501 (28.36)	570 (32.23)	
Diabetes %						< 0.0001
No	6,755 (92.78)	1777 (96.83)	1716 (93,75)	1,653 (91.85)	1,609 (88.12)	
Yes	685(7.22)	82(3.17)	145(6.25)	205(8.15)	253 (11.88)	
Hypertension %						< 0.0001
No	5,448 (74.42)	1,536 (83.55)	1,437 (78.03)	1,296 (70.28)	1,179 (64.49)	
Yes	1992 (25.58)	323 (16.45)	424 (21.97)	562 (29.72)	683 (35.51)	
Cardiovascular diseases %						0.84
No	7,205 (97.50)	1810 (97.79)	1807 (97.56)	1795 (97.23)	1793 (97.40)	
Yes	235(2.50)	49 (2.21)	54 (2.44)	63 (2.77)	69 (2.60)	
Physical activity %						< 0.001
No	1,124 (14.13)	360 (17.50)	278 (14.60)	265 (12.44)	221 (11.54)	
Yes	6,316 (85.87)	1,499 (82.50)	1,583 (85.40)	1,593 (87.56)	1,641 (88.46)	
PIR	3.01 ± 0.05	3.17 ± 0.07	2.92 ± 0.08	2.97 ± 0.08	2.96 ± 0.06	0.002
BMI(kg/m^2^)	28.37 ± 0.13	23.49 ± 0.12	26.91 ± 0.12	29.23 ± 0.15	34.59 ± 0.21	< 0.0001
PhenoAge	35.65 ± 0.33	35.01 ± 0.50	34.93 ± 0.51	36.42 ± 0.39	36.35 ± 0.41	0.01
PhenoAgeAccel	−3.05 ± 0.11	−4.32 ± 0.15	−3.14 ± 0.13	−2.77 ± 0.13	−1.78 ± 0.15	< 0.0001
SII	500.40 ± 5.12	522.85 ± 11.50	501.88 ± 8.35	498.41 ± 10.20	479.17 ± 5.98	0.01
Alkaline phosphatase (U/L)	65.50 ± 0.39	62.86 ± 0.69	66.03 ± 0.55	66.41 ± 0.61	67.05 ± 0.56	< 0.0001
Serum creatinine (mg/dL)	0.86 ± 0.00	0.74 ± 0.01	0.84 ± 0.01	0.91 ± 0.01	0.96 ± 0.01	< 0.0001
Total protein (g/dL)	7.14 ± 0.01	7.09 ± 0.02	7.14 ± 0.02	7.16 ± 0.02	7.16 ± 0.02	0.002
Serum uric acid (mg/dL)	5.35 ± 0.03	4.41 ± 0.03	5.23 ± 0.04	5.71 ± 0.04	6.18 ± 0.04	< 0.0001
Blood urea nitrogen (mg/dL)	12.78 ± 0.09	11.85 ± 0.13	12.32 ± 0.14	13.45 ± 0.15	13.62 ± 0.13	< 0.0001
Serum phosphorus (mg/dL)	3.72 ± 0.01	3.83 ± 0.02	3.69 ± 0.02	3.68 ± 0.02	3.68 ± 0.02	< 0.0001
Serum calcium (mg/dL)	9.38 ± 0.01	9.37 ± 0.01	9.38 ± 0.01	9.40 ± 0.01	9.38 ± 0.01	0.15

Data are expressed as weighted means ± SD or percentages (%). BMI, body mass index, PIR, poverty income ratio, SII, Systemic Inflammation Index, PhenoAge, phenotypic age, PhenoAgeAccel, Phenotypic Age Acceleration.

### 3.2 Association between ASMI and PhenoAgeAccel

Table 2 presents the multivariable regression analysis examining the association between ASMI and PhenoAgeAccel. The results indicate that higher ASMI is associated with a decrease in PhenoAgeAccel. In Model 3, a significant inverse correlation was observed (*β* = −0.48, 95% CI: −0.66 to −0.29, *p* = 0.0001), showing that each unit increase in ASMI corresponded to a reduction of approximately 0.48 years in PhenoAgeAccel. When ASMI was categorized into quartiles, participants in Quartile 4 had significantly lower PhenoAgeAccel compared to those in Quartile 1 (*β* = −0.97, 95% CI: −1.67 to −0.27, *p* = 0.01). Quartile 3 participants also showed a reduction in PhenoAgeAccel (*β* = −0.55, 95% CI: −1.05 to −0.05, *p* = 0.03). These results suggest a robust inverse relationship between ASMI and phenotypic aging, highlighting the potential role of muscle mass in mitigating age-related biological decline.

**Table 2:** Multivariate linear analysis of the association between appendicular skeletal muscle mass index (ASMI) and PhenoAge Acceleration (PhenoAgeAccel).

	β**(95%CI),p-value**		
ASMI	Model 1	Model 2	Model 3
Continuous (per SD)	0.58 (0.51, 0.65) < 0.0001	0.72 (0.62, 0.83) < 0.0001	−0.48(−0.66, −0.29) < 0.0001
Categories			
Q1	Reference	Reference	Reference
Q2	1.17 (0.86, 1.49) < 0.0001	1.56 (1.19, 1.93) < 0.0001	−0.21 (−0.59, 0.16) 0.25
Q3	1.55 (1.21, 1.89) < 0.0001	2.18 (1.70, 2.65) < 0.0001	−0.55 (−1.05, −0.05)0.03
Q4	2.54 (2.20, 2.87) < 0.0001	3.18 (2.67, 3.70) < 0.0001	−0.97 (−1.67, −0.27) 0.01
p for trend	<0.001	<0.001	0.01

Data are presented as β coefficient (95% CI). Model 1 was unadjusted; Model 2 adjusted for gender, age, and race; Model 3 included additional adjustments for marital status, education, poverty income ratio, smoking status, drinking status, physical activity, hypertension, diabetes, cardiovascular diseases, body mass index, and biochemical markers (total protein, blood urea nitrogen, serum creatinine, serum uric acid, serum calcium, alkaline phosphatase, and serum phosphorus).

### 3.3 Association between ASMI and SII

Table 3 presents the multivariable regression results examining the association between ASMI and SII. Our analysis shows a significant negative correlation between ASMI and SII. In Model 3, each unit increase in ASMI was associated with a 28.28 unit decrease in SII (*β* = −28.28, 95% CI: −41.77 to −14.79, *p* < 0.001). When ASMI was categorized into quartiles, participants in the highest ASMI quartile exhibited significantly lower SII levels compared to those in the lowest quartile, with a reduction of 69.3 units (*β* = −69.3, 95% CI: −117.58 to −21.02, *p* = 0.01). This finding suggests a robust inverse relationship between muscle mass and systemic inflammation, underscoring the potential role of higher ASMI in mitigating inflammation-related health risks.

**Table 3:** Multivariate linear analysis of the association between appendicular skeletal muscle mass index (ASMI) and systemic immune-inflammation index(SII).

	*β* (95%CI), *p*-value		
ASMI	Model 1	Model 2	Model 3
Continuous(per SD)	−8.84 (−14.25, −3.43) 0.002	6.69 (0.28, 13.10) 0.04	−28.28 (−41.77, −14.79) < 0.001
Categories			
Q1	Reference	Reference	Reference
Q2	−14.15 (−37.63, 9.34) 0.23	20.57 (−3.54, 44.67) 0.09	−24.87 (−50.72, 0.98) 0.06
Q3	−22.3 (−50.78, 6.17) 0.12	34.06 (5.18, 62.94) 0.02	−32.91 (−68.86, 3.04) 0.07
Q4	−40.72 (−65.58, −15.86) 0.002	30.19 (−0.52, 60.91) 0.05	−69.3 (−117.58, −21.02) 0.01
*p* for trend	0.002	0.05	0.01

Data are presented as β coefficient (95% CI). Model 1 was unadjusted; Model 2 adjusted for gender, age, and race; Model 3 included additional adjustments for marital status, education, poverty income ratio, smoking status, drinking status, physical activity, hypertension, diabetes, cardiovascular diseases, body mass index, and biochemical markers (total protein, blood urea nitrogen, serum creatinine, serum uric acid, serum calcium, alkaline phosphatase, and serum phosphorus).

### 3.4 Association between SII and PhenoAgeAccel

Table 4 shows the multivariable regression analysis results examining the relationship between SII and PhenoAgeAccel. SII was consistently and significantly associated with PhenoAgeAccel across all models. In Model 3, each unit increase in log2-SII was linked to an approximate 2.39-year increase in PhenoAgeAccel (*β* = 2.39, 95% CI: 2.24 to 2.55, *p* < 0.0001). When SII was categorized into quartiles, participants in the highest SII quartile exhibited significantly higher PhenoAgeAccel compared to those in the lowest quartile (all *p* < 0.0001). This strong positive association highlights the potential role of systemic inflammation in accelerating biological aging.

**Table 4:** Multivariate linear analysis of the association between systemic immune-inflammation index (SII) and PhenoAge Acceleration (PhenoAgeAccel).

	*β* (95%CI), *p*-value		
SII	Model 1	Model 2	Model 3
Log2-transformed SII	2.53 (2.33, 2.73) < 0.0001	2.77 (2.58, 2.96) < 0.0001	2.39 (2.24, 2.55) < 0.0001
Categories			
Q1	Reference	Reference	Reference
Q2	1.05 (0.78, 1.32) < 0.0001	1.3 (1.05, 1.55) < 0.0001	1.16 (0.95, 1.36) < 0.0001
Q3	2.48 (2.09, 2.88) < 0.0001	2.81 (2.44, 3.19) < 0.0001	2.4 (2.09, 2.71) < 0.0001
Q4	4.44 (4.11, 4.77) < 0.0001	4.84 (4.53, 5.15) < 0.0001	4.18 (3.92, 4.44) < 0.0001
*p* for trend	<0.0001	<0.0001	<0.0001

Data are presented as β coefficient (95% CI). Model 1 was unadjusted; Model 2 adjusted for gender, age, and race; Model 3 included additional adjustments for marital status, education, poverty income ratio, smoking status, drinking status, physical activity, hypertension, diabetes, cardiovascular diseases, body mass index, and biochemical markers (total protein, blood urea nitrogen, serum creatinine, serum uric acid, serum calcium, alkaline phosphatase, and serum phosphorus).

### 3.5 Mediation analyses

**Figure 2:**
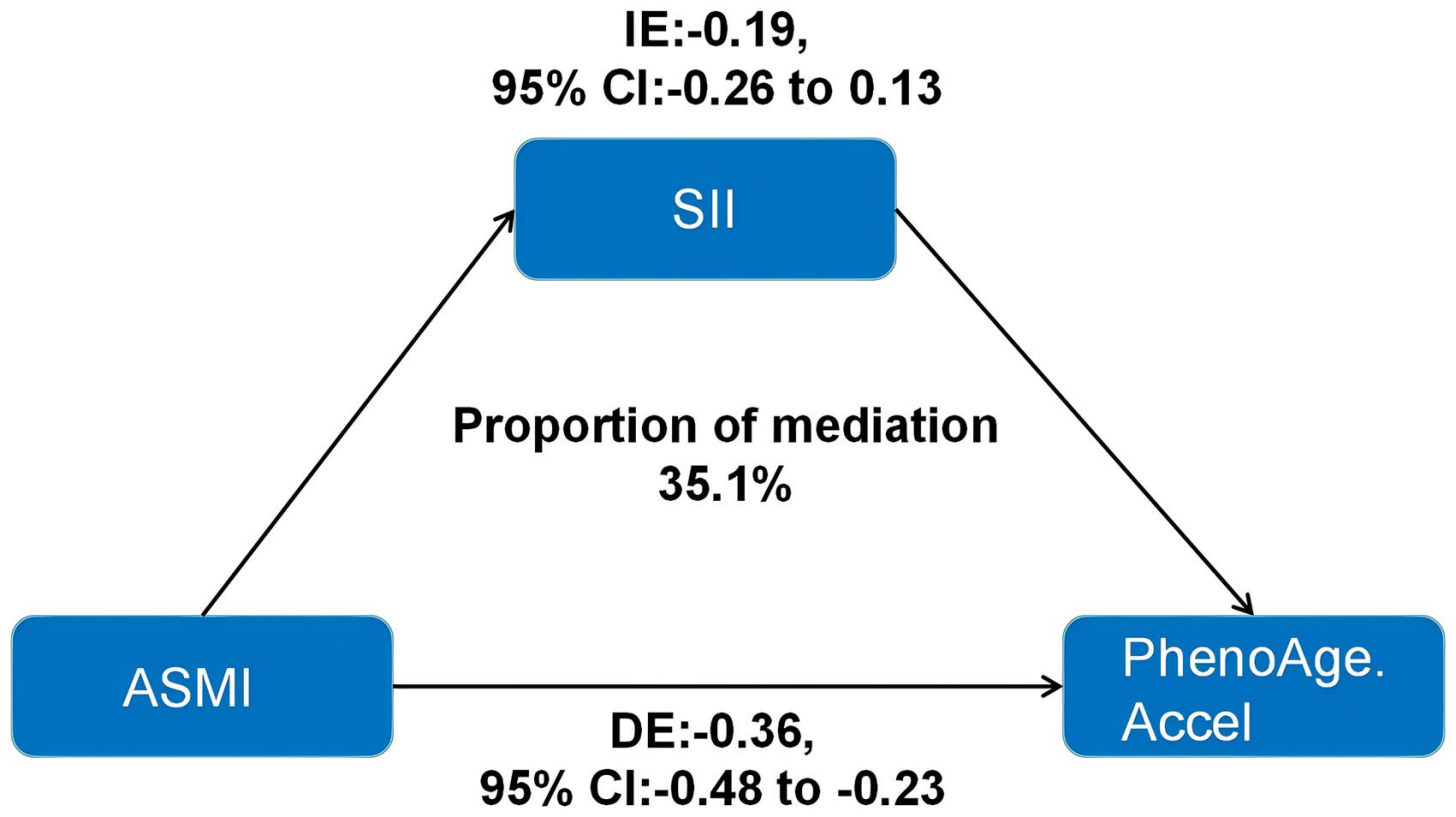
Indirect effects of inflammation indicators in the association between ASMI and PhenoAge.Accel. ASMI: appendicular skeletal muscle mass index; SII: systemic immune-inflammation index; IE: indirect effects; DE: direct effects; PhenoAge.Accel: PhenoAge Acceleration.

Mediation analysis revealed that SII significantly mediated the relationship between ASMI and PhenoAgeAccel, with a mediation proportion of 35.1% (Figure 2). This suggested that systemic inflammation may be a key mechanism linking lower ASMI to accelerated biological aging. These findings underscore the potential importance of addressing inflammation to mitigate the health risks associated with reduced muscle mass and aging.

### 3.6 Subgroup

**Figure 3:**
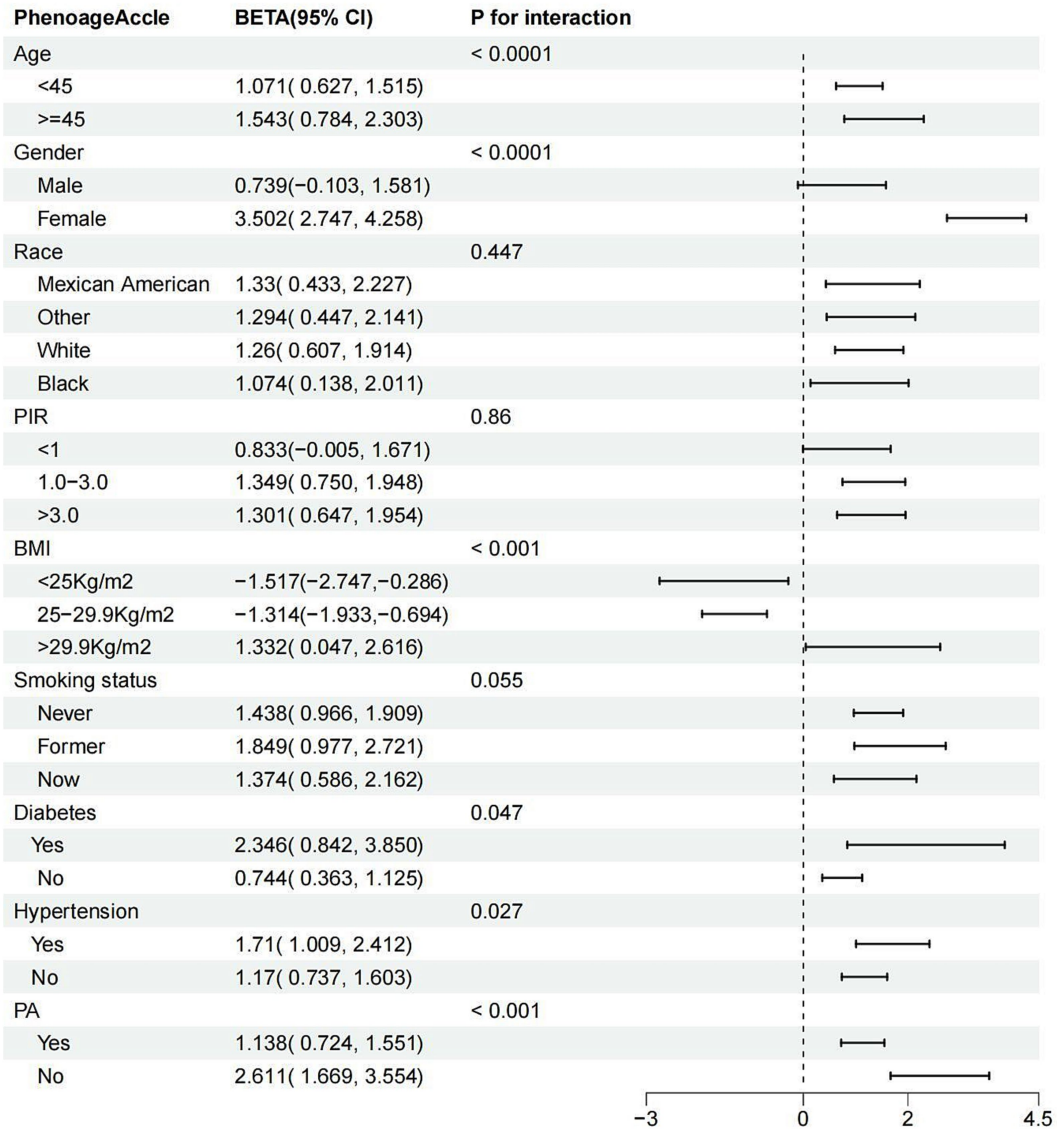
Subgroup analysis for the association between ASMI and PhenoAge.Accel. ASMI: appendicular skeletal muscle mass index; PhenoAge.Accel: PhenoAge Acceleration; PIR: poverty income ratio; PA: physical activity; BMI: body mass index.

Subgroup analysis revealed variability in the association between ASMI and PhenoAgeAccel across different subgroups (Figure 3). A significant relationship was consistently found in subgroups stratified by age, race, smoking status, diabetes, hypertension, and PA, with all *p*-values below 0.05. Interestingly, when stratified by gender, the association was significant only among females (*β* = 3.502; 95% CI: 2.747 to 4.258, *p* < 0.0001). In PIR-stratified subgroups, the relationship was significant only among participants with PIR >1.1 (all *p*-values <0.001). In BMI-stratified subgroups, a negative association between ASMI and PhenoAgeAccel was observed for participants with a BMI below 29.9 kg/m^2^ (<25 kg/m^2^: *β* = −1.517; 95% CI: −2.747 to −0.286, *p* = 0.017; 25–29.9 kg/m^2^: *β* = −1.314; 95% CI: −1.933 to −0.694, *p* < 0.001). Conversely, a positive association was found for participants with a BMI above 29.9 kg/m^2^ (*β* = 1.332; 95% CI: 0.047 to 2.616, *p* = 0.043). Significant interactions were detected with age, gender, BMI, hypertension, diabetes, and PA (all interaction *p*-values <0.05), but no significant interactions were noted for race, PIR, or smoking status.

In summary, the relationship between ASMI and PhenoAgeAccel appears to be influenced by factors such as gender, BMI, hypertension, diabetes, and PA, with the association being more pronounced in females, individuals with obesity, those with diabetes or hypertension, and those with lower physical activity levels.

### 3.7 Restricted cubic spline

**Figure 4:**
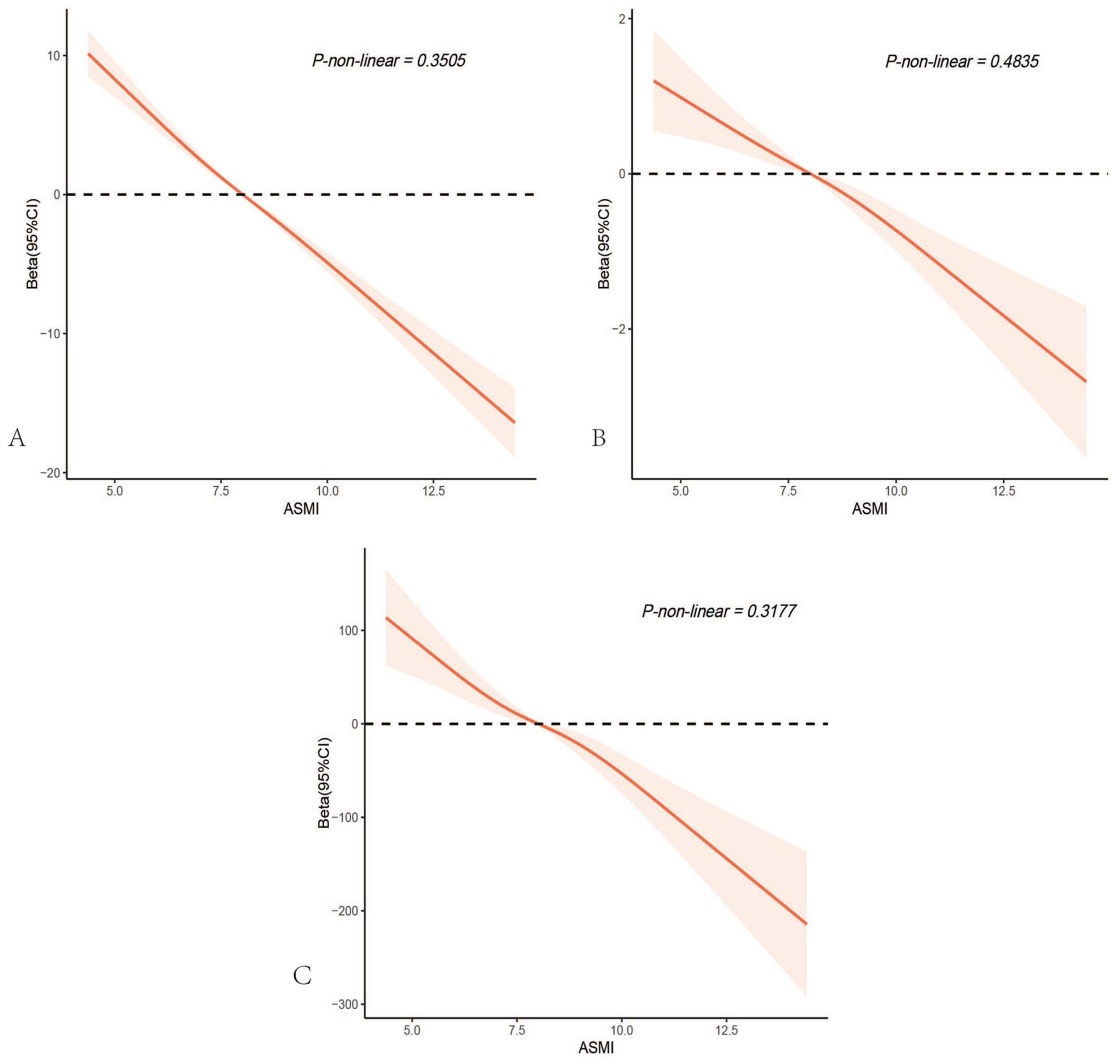
Restricted cubic spline analysis depicting the potential nonlinear associations between appendicular skeletal muscle mass index and age**(A)**, PhenoAge Acceleration **(B)**, and the systemic immune-inflammation index **(C)**. All *p*-values for nonlinearity were > 0.05, indicating no significant evidence of nonlinear associations.

After accounting for multiple covariates, there were no nonlinear associations between ASMI and age, PhenoAgeAccle, or SII (All P-non-linear>0.05). As ASMI increased, age, PhenoAgeAccle, and SII all showed a gradual decline (Figure 4).

## 4 Discussion

This study is the first to examine the relationship between muscle mass, as measured by ASMI, and PhenoAge in a nationally representative population. Our findings reveal a significant association, with higher ASMI scores linked to slower PhenoAge progression. Mediation analysis underscores the role of systemic inflammation in this relationship, suggesting that maintaining muscle mass may help slow biological aging.

To our knowledge, no prior studies have explored this association. Our research is the first to establish a connection between ASMI and PhenoAgeAccel, and to investigate the mediating effect of SII. PhenoAge captures not only disease morbidity but also subclinical aging and mortality risk, providing a more immediate assessment of intervention outcomes (28, 29). Integrating PhenoAge with biological markers offers a comprehensive method for predicting mortality risk (30).

Sarcopenia is a key factor in functional decline among older adults (31). Previous studies have focused on sarcopenia and all-cause mortality, but few have examined the link between muscle mass and biological age. Our results fill this gap, showing that lower ASMI is significantly associated with increased PhenoAgeAccel, consistent with existing evidence linking low muscle mass to higher mortality rates and functional decline(HR = 3.18, 95% CI: 1.53–6.65), (32–34).

Our mediation analysis suggests that chronic inflammation plays a crucial role in the ASMI-PhenoAgeAccel relationship. Individuals with low muscle mass often have elevated levels of pro-inflammatory markers such as IL-6 and TNF-*α*, which accelerate muscle loss and aging (35, 36). Decreasing muscle mass can lead to increased visceral fat, releasing inflammatory factors that promote cellular aging through DNA damage and mitochondrial dysfunction (37, 38). In a study of 270 patients with biopsy-confirmed myopathies, the incidence of inflammatory myopathies was significantly higher in patients aged 70 and above compared to younger patients, indicating that chronic inflammation is closely associated with sarcopenia in the elderly (39).

Clinically, our findings suggest that maintaining or increasing muscle mass may help slow biological aging. Resistance training, aerobic exercise, and caloric restriction have all been shown to improve ASMI, enhance insulin sensitivity, and reduce inflammation (40). Anti-inflammatory interventions, such as NSAIDs, may also help reduce inflammation and prevent muscle loss in older adults (41, 42). We recommend a combination of muscle-strengthening exercises, anti-inflammatory diets, and pharmacological interventions to mitigate PhenoAgeAccel and reduce age-related health risks.

The strengths of this study include the use of nationally representative data and a large sample size, which enhance the generalizability of our findings. Additionally, we not only revealed a negative correlation between ASMI and PhenoAgeAccel but also explored the role of chronic inflammation through mediation analysis, providing new insights into the mechanisms of aging. We also adjusted for multiple confounding factors to ensure the robustness of the results.

However, this study has several limitations. First, although PhenoAgeAccel is a validated indicator of biological age acceleration, it is not the only standard. Future studies could explore the roles of other biological markers in the aging process. Second, the cross-sectional design captures associations at a single time point and cannot assess causal relationships. Therefore, longitudinal studies are needed to more deeply verify the causal effect of muscle mass on biological age acceleration. Third, this study focused on chronic inflammation as assessed by SII, which provides a robust measure of systemic inflammation. However, our study lacks clear thresholds or supplementary inflammatory markers to distinguish subtypes of chronic inflammation, such as chronic low-grade inflammation. Future research should aim to incorporate a wider range of markers to better characterize inflammation subtypes. Lastly, despite adjusting for multiple known confounding factors, residual confounding may still exist, especially unmeasured variables related to lifestyle and diet, which could influence the study results.

## 5 Conclusion

In U.S. adults, higher ASMI scores were significantly associated with delayed biological aging. Additionally, our findings suggest that inflammation partially mediates the relationship between ASMI and PhenoAgeAccel, offering important insights into the biological mechanisms linking muscle mass and the aging process. These results emphasize the critical role of maintaining muscle mass in mitigating age-related biological decline. This has substantial public health implications, particularly in designing targeted interventions aimed at slowing aging and improving overall health outcomes, such as resistance training programs, anti-inflammatory diets, and personalized medical treatments.

## Data Availability

Publicly available datasets were analyzed in this study. This data can be found at: http://www.cdc.gov/nchs/nhanes.htm.

## Footnotes

^1^ http://www.cdc.gov/nchs/nhanes.htm
